# Hypoattenuation Pattern on Contrast-Enhanced Computed Tomography Predicts Poor Prognosis in Patients with Pancreatic Neuroendocrine Tumors

**DOI:** 10.3390/jcm15062252

**Published:** 2026-03-16

**Authors:** Issei Kojima, Shinichi Hashimoto, Yu Yamazato, Ryusuke Shibata, Yusuke Kamikihara, Koshiro Toyodome, Takafumi Hamada, Makoto Hinokuchi, Shiho Arima, Shiroh Tanoue, Fumisato Sasaki, Fumitaka Ejima, Koji Takumi, Takashi Yoshiura, Shuji Kanmura

**Affiliations:** 1 Digestive and Lifestyle Diseases, Kagoshima University Graduate School of Medical and Dental Sciences, 8-35-1 Sakuragaoka, Kagoshima 890-8520, Japan; isseikjm8603@gmail.com (I.K.); yu.yamazato@gmail.com (Y.Y.); ryusukeshibata0519@gmail.com (R.S.); k.yusuke0024@gmail.com (Y.K.); koshirotoyodome@gmail.com (K.T.); m07078th@gmail.com (T.H.); m.hinokuchi8013@gmail.com (M.H.); sarima@m3.kufm.kagoshima-u.ac.jp (S.A.); tanoue@m.kufm.kagoshima-u.ac.jp (S.T.); bungohs@m2.kufm.kagoshima-u.ac.jp (F.S.); skanmura@m2.kufm.kagoshima-u.ac.jp (S.K.); 2 Department of Radiology, Kagoshima University Graduate School of Medical and Dental Sciences, 8-35-1 Sakuragaoka, Kagoshima 890-8520, Japan; k8716799@kadai.jp (F.E.); takumi@m2.kufm.kagoshima-u.ac.jp (K.T.); yoshiura@m3.kufm.kagoshima-u.ac.jp (T.Y.)

**Keywords:** pancreatic neuroendocrine tumor, contrast-enhanced computed tomography, hyperattenuation pattern, overall survival, progression-free survival

## Abstract

**Background/Objectives:** Several reports have shown that the hypoattenuation pattern of contrast-enhanced computed tomography (CECT) in pancreatic neuroendocrine tumors (PanNETs) is associated with poor overall survival (OS). However, these studies also included neuroendocrine carcinoma. Therefore, this study retrospectively investigated the relationship between attenuation patterns and OS, specifically in PanNETs. **Methods:** Between July 2005 and August 2022, 80 consecutive patients (median age, 64 years; 39 males and 41 females) with pathologically confirmed PanNETs were enrolled. Pretreatment factors associated with PanNET prognosis were evaluated. **Results:** The median tumor diameter was 18 mm (range, 6–150 mm). The PanNET grades were G1 in 45 patients (56%), G2 in 31 (39%), and G3 in 4 (5%). Hyperattenuation and hypoattenuation patterns were observed in 64 (80%) and 16 (20%) patients, respectively. Surgery was performed on 63 patients (79%), and 18 (23%) had distant metastases. Multivariate analysis identified the hypoattenuation pattern on pretreatment CECT as a factor significantly associated with OS in all participants (*p* < 0.001; hazard ratio [HR], 9.45) and in patients with PanNET G2 (*p* = 0.007; HR, 9.13). Median OS was longer in the hyperattenuation group than in the hypoattenuation group among all participants (hyperattenuation, not reached; hypoattenuation, 40.1 months; *p* < 0.001) and among patients with PanNET G2 (hyperattenuation, not reached; hypoattenuation, 36.3 months; *p* = 0.001). **Conclusions:** PanNETs with a hypoattenuation pattern on CECT had a worse prognosis than those with hyperattenuation, even among tumors of the same pathological grade (G2).

## 1. Introduction

Pancreatic neuroendocrine neoplasms (PanNENs) are rare, accounting for 1–5% of all pancreatic malignancies [1,2]. In the 2017 World Health Organization (WHO) classification, PanNENs were categorized into four pathological types: pancreatic neuroendocrine tumor (PanNET) grade (G) 1, G2, G3, and pancreatic neuroendocrine carcinoma (PanNEC), according to tumor proliferative ability (Ki-67 proliferation index and mitotic rate) and malignant differentiation. Each PanNET grade exhibits similar biological behavior and treatment strategies. However, PanNEC is frequently associated with distant metastasis and poor prognosis, requiring distinct treatment approaches [3].

The incidence of PanNENs has increased in recent years owing to advances in imaging modalities [4]. Dynamic contrast-enhanced computed tomography (CECT) is a useful modality for detecting and staging PanNENs [5]. PanNETs typically appear as well-circumscribed, hyperattenuating solid masses on pancreatic-phase images. However, some PanNETs demonstrate hypoattenuation compared to the normal pancreatic parenchyma during the pancreatic phase [6]. A hypoattenuation pattern on computed tomography (CT) has been reported to be associated with a higher malignant potential in PanNENs [6,7]. These reports included PanNEC because, under the previous WHO classification, PanNET G3 and PanNEC were grouped. Few studies have evaluated the relationship between CECT enhancement patterns and survival outcomes, specifically in PanNETs.

We retrospectively evaluated the correlation between CECT enhancement patterns and the prognosis of PanNETs of all types and grades, excluding PanNECs.

## 2. Materials and Methods

### 2.1. Patient Population

A total of 124 patients were diagnosed with PanNENs by imaging or histology at our hospital between July 2005 and August 2022. Imaging findings and clinical outcomes were retrospectively reviewed from clinical records and electronic databases. Patients were excluded for the following reasons: lack of CECT before treatment (*n* = 22), presence of PanNEC (*n* = 9), unavailability of pathological specimens for grading based on the 2017 WHO classification (*n* = 7), diagnosis of mixed neuroendocrine-non-neuroendocrine neoplasms (*n* = 1), and observation period <30 days (*n* = 5). Eighty patients were included in the study. Among them, 71 had unifocal PanNENs, and 9 had multifocal PanNENs. For the nine patients with multifocal cases, the largest tumor was selected as the representative lesion.

This study was conducted in accordance with the 2013 Declaration of Helsinki and was approved by the Institutional Review Board of Kagoshima University Hospital (230093). Informed consent was obtained from all patients in the form of an opt-out.

#### Diagnosis and Confirmation of the Grading of PanNET

All PanNETs and tumor grades were pathologically diagnosed. Final diagnoses were confirmed by pathologists specializing in the pancreatobiliary system using surgical samples (*n* = 63) or biopsy samples obtained via endoscopic ultrasound-guided tissue acquisition (EUS-TA) (*n* = 17). Of the patients diagnosed with EUS-TA who did not undergo surgery, 12 underwent fine-needle aspiration, and five underwent fine-needle biopsy. Two cases were diagnosed during the second EUS-TA session. Seventy-three cases of PanNETs were reviewed according to the 2017 WHO classification and categorized as G1, G2, or G3 based on pathological findings, Ki-67 labeling index, and mitotic activity, and the remaining 7 cases (G1, *n* = 1; G2, *n* = 4; G3, *n* = 2) were mainly done according to the Ki-67 labeling index. Cases with inadequate specimens for grading were excluded from the study.

### 2.2. Imaging Protocol

Several imaging modalities, including CT, were used for tumor staging. A radiologist specializing in gastroenterology determined the tumor location, size (longest diameter), and stage according to the 8th edition of the Union for International Cancer Control (UICC) classification of PanNEN.

CT examinations were performed using a 16- or 64-multidetector row CT scanner (Aquilion, Canon Medical Systems, Tochigi, Japan, or IQon spectral CT, Philips Healthcare, Best, the Netherlands). Multiphase CECT (unenhanced, pancreatic parenchymal, portal venous, and delayed phases) was routinely performed to evaluate pancreatic tumors. The imaging parameters for all phases were as follows: tube voltage, 120 kVp; gantry rotation speed, 0.4 or 0.5 s; pitch, 0.703 or 0.828; maximum allowable tube current, 440 mA; and detector row configuration of 16 mm × 1 mm (*n* = 13), 64 mm × 0.5 mm (*n* = 43), or 64 × 0.625 mm (*n* = 24). The scan delays for the pancreatic and portal venous phases were 20 s and 48 s, respectively, after aortic enhancement exceeded 150 Hounsfield units (HU) relative to baseline. The scan delay for the delayed phase was fixed at 180 s after intravenous injection of 1.7 mL/kg body weight of nonionic contrast material (iodine concentration, 350 mgI/mL; Iomeron; Eisai, Tokyo, Japan) administered over 25 s.

**Figure 1:**
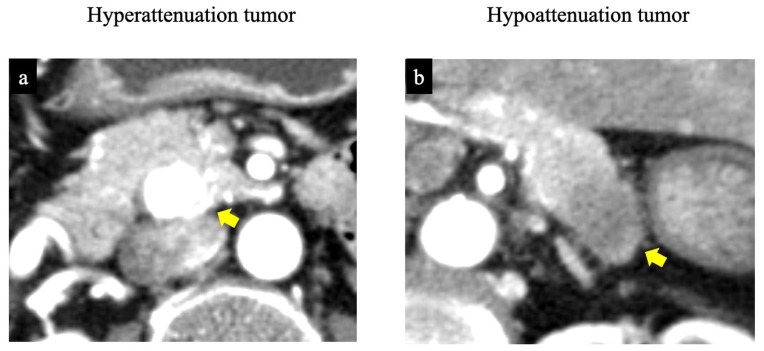
Findings of contrast-enhanced computed tomography: (**a**) Hyperattenuated tumor (arrow) showing higher tumor attenuation than the pancreatic parenchyma (Hounsfield unit ratio of tumor to pancreatic parenchyma during the pancreatic parenchymal phase ≥1). (**b**) Hypoattenuated tumor (arrow) showing lower attenuation than the pancreatic parenchyma (Hounsfield unit ratio during the pancreatic phase <1).

Based on pretreatment CECT findings, the tumors were classified into hyperattenuation and hypoattenuation groups. HU values of the tumor area and pancreatic parenchyma were measured during the pancreatic parenchymal phase. The hyperattenuation group was defined as a ratio of tumor HU to pancreatic parenchyma HU ≥1.0, and the hypoattenuation group as a ratio of <1.0 (Figure 1). HU measurements for all PanNETs were performed by radiologists who did not participate in the qualitative imaging analysis and were blinded to the pathological tumor grades. A circular or oval region of interest, as large as possible, was placed at identical sites within each tumor and the pancreatic parenchyma to avoid calcification and cystic components.

Patients who underwent curative surgery were routinely monitored for recurrence using imaging. For cases with recurrence or inoperable disease, tumor response to therapy was assessed according to the Response Evaluation Criteria in Solid Tumors using CT at 3 months. Overall survival (OS) was defined as the interval from the initial CECT assessment to death or the last follow-up.

### 2.3. Statistical Analysis

Variables between the two groups were analyzed using the Mann–Whitney U test, chi-squared test, Fisher’s exact test, and log-rank test, as appropriate. Statistical significance was set at *p* < 0.05. To avoid overfitting, variables with *p* < 0.01 in the univariate analyses were entered into a Cox proportional hazards model using the stepwise method to identify factors associated with OS. The Kaplan–Meier method with the log-rank test was used to estimate OS and progression-free survival (PFS) in the hyperattenuation and hypoattenuation groups. All statistical analyses were performed using EZR version 1.55 (Saitama Medical Center, Jichi Medical University, Saitama, Japan), a graphical user interface for R (The R Foundation for Statistical Computing, version 4.1.2, Vienna, Austria).

## 3. Results

### 3.1. Patient Characteristics for All Participants

The baseline characteristics of the patients are summarized in Table 1. Functional PanNETs were present in 23 cases (insulinoma, *n* = 14; gastrinoma, *n* = 5; glucagonoma, *n* = 4). There were six MEN-1 cases, four of which had multiple pancreatic tumors, and two had a single pancreatic tumor. The median tumor diameter was 18 (range, 6–150) mm. Of all patients, 45 were classified as PanNET G1 (56%), 31 as PanNET G2 (39%), and four as PanNET G3 (5%). The pathological characteristics according to each tumor grade are shown in Supplementary Table S1. The median (range) Ki-67 labeling indices were 1.0 (0–2.8) % in PanNET G1, 5.0 (3.0–18.0) % in PanNET G2, and 35.0 (30.0–50.0) % in PanNET G3.

**Figure 2:**
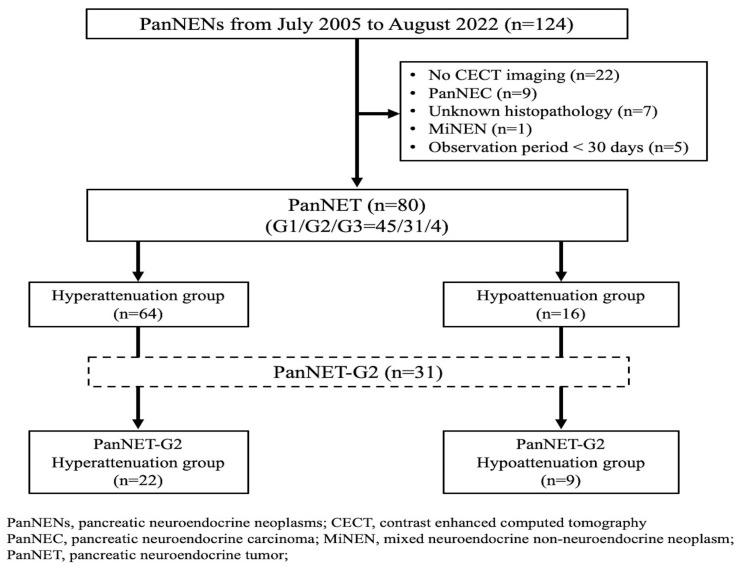
Flowchart of the study population of patients with pancreatic neuroendocrine tumors.

Sixty-four (80%) and 16 (20%) patients were classified into the hyperattenuation and hypoattenuation groups, respectively (Figure 2). In all patients, the median (range) HU value of CECT for the tumor and the pancreatic parenchyma was 205.12 (61.82–440.86) (hyperattenuation group, 217.31 [108.25–440.86]; hypoattenuation group, 118.36 [61.82–157.22], *p* < 0.001) and 157.29 (95.24–213.69) (hyperattenuation group, 157.29 [95.24–205.28]; hypoattenuation group, 158.44 [105.66–213.69], *p* = 0.732), respectively (Supplementary Table S2). The median (range) HU ratio of tumor to pancreatic parenchyma was 1.31 [0.38–3.32] (hyperattenuation group, 1.43 [1.00–3.32]; hypoattenuation group, 0.75 [0.38–0.99], *p* < 0.001). To evaluate whether HU values changed over the long-term study period or as a result of the CECT protocol and modality, the HU values of the tumor and the tumor-to-pancreatic parenchyma HU ratio were compared between the first and last 40 cases, yielding similar results (Supplementary Table S3). The median tumor size was significantly smaller in the hyperattenuation group than in the hypoattenuation group (15 vs. 33 mm; *p* < 0.001). The rates of heterogeneous contrast pattern (53% vs. 88%, *p* = 0.020) and main pancreatic duct dilation ≥5 mm (3% vs. 25%, *p* = 0.013) were also lower in the hyperattenuation group.

The hypoattenuation group had significantly higher rates of metastasis (13% vs. 63%, *p* < 0.001) and a higher median Ki-67 labeling index (1.9% vs. 6.5%, *p* = 0.002). The pathological grade distribution differed significantly between the two groups (hyperattenuation: G1/G2/G3 = 64%/34%/2%; hypoattenuation: 25%/56%/19%; *p* = 0.003). The distribution of UICC stages was more advanced in the hypoattenuation group (I/II/III/IV = 13%/25%/6%/56%) than in the hyperattenuation group (I/II/III/IV = 56%/30%/5%/9%) (*p* < 0.001). In cases where intratumoral necrosis could be pathologically evaluated (the total cases, *n* = 34; 10.0% vs. 50.0%, *p* = 0.094; cases who underwent surgery, *n* = 32; 10.3% vs. 66.7%; *p* = 0.057), the incidence of pathological intratumoral necrosis tended to be higher in the hypoattenuation group than in the hyperattenuation group (Supplementary Table S4).

### 3.2. Clinical Outcomes of All Cases with PanNET

The median follow-up period for the entire cohort was 50.9 months (range, 1.9–163.1 months). Regarding primary treatment, 63 patients underwent surgery (curative intent, *n* = 57; debulking intent, *n* = 6), seven received drug therapy, two underwent peptide receptor radionuclide therapy (PRRT), and eight received no treatment. Among the 63 surgical cases, postoperative recurrence occurred in eight patients (two after curative-intent surgery and six after debulking-intent surgery). Recurrences were predominantly liver metastases (*n* = 7), with one patient developing cardiac and bone metastases. Treatments for recurrence included transcatheter arterial chemoembolization (TACE) (*n* = 5), drug therapy (*n* = 4), and radiotherapy (*n* = 2). During follow-up, 12 patients died (hyperattenuation group, *n* = 3; hypoattenuation group, *n* = 9). Tumor-related deaths occurred in eight patients (median, 10.4 months; range, 3.3–40.8 months; hyperattenuation group, *n* = 1; hypoattenuation group, *n* = 7). The causes of death in the remaining four cases were myocardial infarction (two cases), Clostridium difficile enteritis, and senility.

The results of the univariate and multivariate analyses of OS in patients with PanNETs are presented in Table 2. In the multivariate analysis, PanNET G2-3 (hazard ratio [HR], 9.48; 95% confidence interval [CI], 1.18–76.06; *p* = 0.034) and the hypoattenuation pattern on CECT (HR, 9.45; 95% CI, 2.49–35.81; *p* < 0.001) were significantly associated with worse OS. Furthermore, multivariate analysis revealed the hypoattenuation pattern on CECT (HR, 5.38; 95% CI, 1.03–28.08; *p* = 0.046) and debulking surgery (HR, 6.02; 95% CI, 1.17–31.14; *p* = 0.032) as factors significantly associated with OS in patients who underwent surgery (Supplementary Table S5).

**Figure 3:**
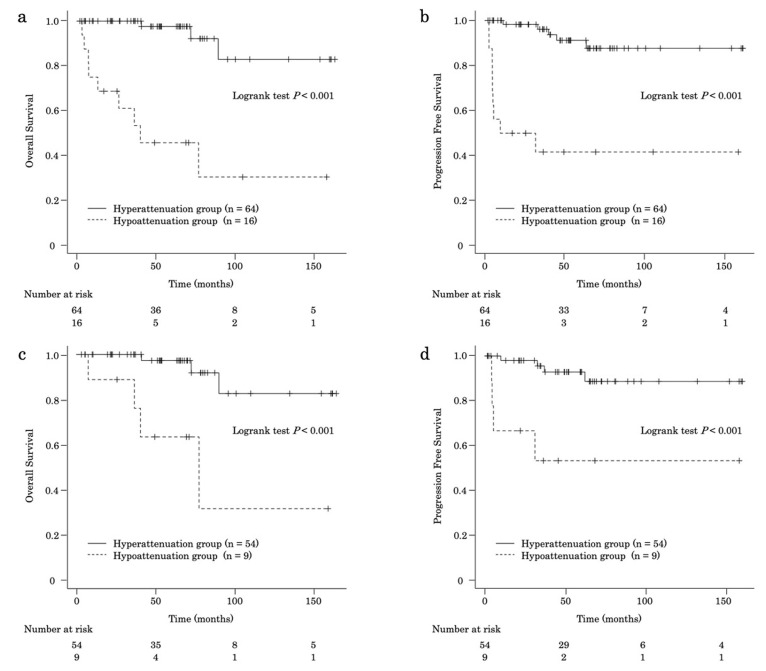
(**a**) Kaplan–Meier curve of overall survival in the hyperattenuation and hypoattenuation groups of patients. (**b**) Kaplan–Meier curve of progression-free survival in the hyperattenuation and hypoattenuation groups. (**c**) Kaplan–Meier curve of overall survival in the hyperattenuation and hyperattenuation groups among patients who underwent surgery. (**d**) Kaplan–Meier curve of progression-free survival in the hyperattenuation and hyperattenuation groups among patients who underwent surgery.

The median OS was longer in the hyperattenuation group than in the hypoattenuation group (hyperattenuation, not reached; hypoattenuation, 40.1 months [95% CI, 7.49–NA]; *p* < 0.001) (Figure 3a). The OS rates at 1, 3, 5, and 10 years were 100%, 100%, 97.6%, and 82.9%, respectively, in the hyperattenuation group and 75.0%, 61.1%, 45.8%, and 30.6%, respectively, in the hypoattenuation group (Supplementary Table S6). The median PFS was also longer in the hyperattenuation group (hyperattenuation, not reached; hypoattenuation, 20.6 months [95% CI, 4.67–NA]; *p* < 0.001) (Figure 3b). The PFS rates at 1, 3, 5, and 10 years were 98.2%, 96.1%, 91.1%, and 87.6%, respectively, in the hyperattenuation group and 50%, 41.7%, 41.7%, and 41.7%, respectively, in the hypoattenuation group.

In patients who underwent surgery (*n* = 63), the OS rates at 1, 3, 5, and 10 years were better in the hyperattenuation group (*n* = 54) than in the hypoattenuation group (*n* = 9) (hyperattenuation: 100%, 100%, 97.3%, and 82.7%, respectively; hypoattenuation: 88.9%, 88.9%, 63.5%, and 31.7%, respectively; *p* < 0.001) (Figure 3c). In addition, the PFS rates were higher in the hyperattenuation group (98.0%, 95.5%, 92.9%, and 88.6%, respectively) than in the hypoattenuation group (66.7%, 53.3%, 53.3%, and 53.3%, respectively) (*p* < 0.001) (Figure 3d).

### 3.3. Patient Characteristics and Clinical Outcomes of Cases with PanNET G2

The baseline characteristics of the 31 patients with PanNET G2 are summarized in Table 3. Twenty-two patients were in the hyperattenuation group and nine in the hypoattenuation group. The groups were similar in most variables, except for tumor size, metastasis, UICC stage, and resection rate.

Univariate and multivariate analyses of OS in patients with PanNET G2 are summarized in Table 4. Univariate analysis identified non-resection (HR, 4.61; 95% CI, 1.13–18.79; *p* = 0.033) and hypoattenuation pattern on CECT (HR, 9.13; 95% CI, 1.83–45.51; *p* = 0.007) as independent factors associated with worse OS. Multivariate analysis revealed that the hypoattenuation pattern on CECT (HR, 9.13; 95% CI, 1.83–45.51; *p* = 0.007) was an independent factor associated with worse OS.

**Figure 4:**
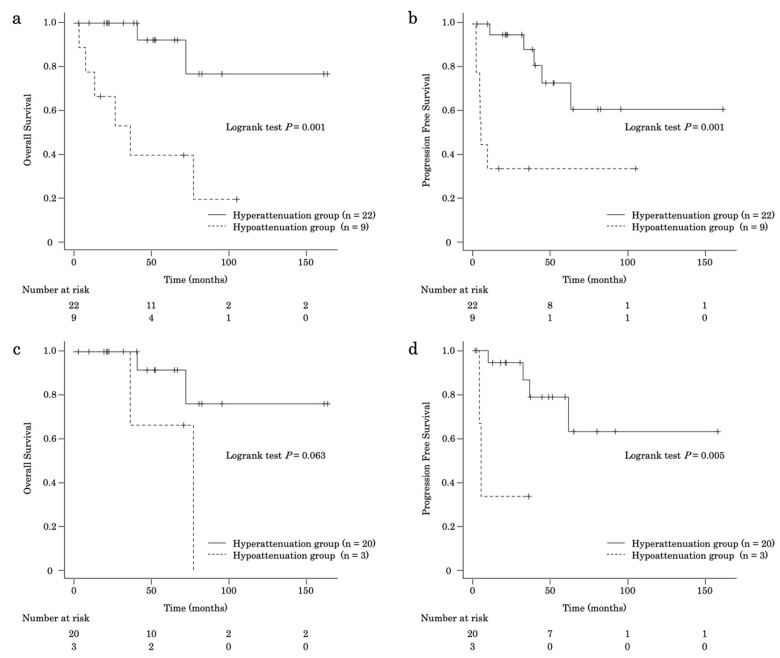
(**a**) Kaplan–Meier curve of overall survival in the hyperattenuation and hypoattenuation groups of patients with grade 2 pancreatic neuroendocrine tumors. (**b**) Kaplan–Meier curve of progression-free survival in the hyperattenuation and hypoattenuation groups for grade 2 tumors. (**c**) Kaplan–Meier curve of overall survival time in the hyperattenuation and hypoattenuation groups among patients with grade 2 tumors who underwent surgery. (**d**) Kaplan–Meier curve of progression-free survival in the hyperattenuation and hypoattenuation groups among patients with grade 2 tumors who underwent surgery.

The median OS in patients with PanNET G2 was shorter in the hypoattenuation group than in the hyperattenuation group (hyperattenuation group, not reached; hypoattenuation group, 36.3 months [95% CI, 3.3–NA]; *p* = 0.001) (Figure 4a). In patients with PanNET G2, the OS rates at 1, 3, 5, and 10 years were 100%, 100%, 92.3%, and 76.9%, respectively, in the hyperattenuation group and 77.8%, 53.3%, 40.0%, and 0%, respectively, in the hypoattenuation group. In addition, the median PFS duration in patients with PanNET G2 was shorter in the hypoattenuation group than in the hyperattenuation group (hyperattenuation group, not reached; hypoattenuation group, 5.5 months [95% CI, 2.50–NA]; *p* = 0.001) (Figure 4b). The PFS rates at 1, 3, 5, and 10 years were 95.0%, 88.2%, 72.8%, and 60.6%, respectively, in the hyperattenuation group and 33.3%, 33.3%, 33.3%, and 0%, respectively, in the hypoattenuation group.

In patients with PanNET G2 who underwent surgery (*n* = 23), the OS rates in the hypoattenuation group (*n* = 3) at 1, 3, 5, and 10 years tended to be worse than those in the hyperattenuation group (*n* = 20) (hyperattenuation group, 100%, 100%, 91.7%, and 76.4%, respectively; hypoattenuation group, 100%, 100%, 66.7%, and 0%, respectively; *p* = 0.063) (Figure 4c). The PFS rates in the hyperattenuation group at 1, 3, 5, and 10 years were higher than those in the hypoattenuation group (hyperattenuation group: 94.4%, 86.6%, 78.7%, and 63.0%, respectively; hypoattenuation group: 33.3%, 33.3%, 0%, and 0%, respectively; *p* = 0.005) (Figure 4d).

### 3.4. Patient Characteristics and Clinical Outcomes of Cases with PanNET G1 and G3

Subgroup analyses of the factors affecting OS were conducted for PanNET G1 and G3. In the cases of PanNET G1 (*n* = 45), 41 cases were in the hyperattenuation group, and four cases were in the hypoattenuation group. All patient characteristics (age, sex, tumor functionality, location, number of tumors, distant metastasis, Ki-67 LI, UICC stage, and resection) were similar. Regarding treatment, 38 patients underwent surgery, one underwent PRRT, and six did not receive treatment. All surgical cases were curatively resected without recurrence. PRRT was administered to the patient with liver metastases, and long-term stable disease was achieved. None of the untreated patients showed disease progression. Regarding OS, only one patient in the hyperattenuation group died of myocardial infarction, and there was no significant difference between the groups.

Among the cases of PanNET G3 (*n* = 4), one case was in the hyperattenuation group, and three cases were in the hypoattenuation group. The hyperattenuation group (*n* = 1) was treated with medication and maintained a long-term partial response (OS: 41.4 months). Two patients in the hypoattenuation group (*n* = 3) underwent surgical treatment but experienced postoperative recurrence leading to death (OS; 40.7 and 19.3 months). The remaining patient worsened soon after drug therapy and died within 3.0 months.

## 4. Discussion

We demonstrated that the hypoattenuation pattern of tumors during the pancreatic parenchymal phase on CECT predicted poor prognosis in patients with PanNETs, particularly PanNET G2.

PanNETs have a highly variable prognosis, which is generally more favorable than that of pancreatic adenocarcinoma owing to their biological heterogeneity [8,9]. Complete surgical resection offers the best chance of cure for localized disease [10]. The prognosis is often more favorable for symptomatic functional tumors diagnosed at an early stage [10] and for well-differentiated (G1/G2) tumors, which have a more indolent course than poorly differentiated (G3) tumors [11,12]. Despite the presence of metastatic disease, many PNETs progress slowly, allowing prolonged survival with advanced therapies.

Recent developments in imaging technology, including CECT, have contributed to the improved detection and staging of PanNENs [13]. Advances in the acquisition of pathological samples have also influenced treatment strategies for PanNENs. EUS-TA enables the collection of histological samples required for immunostaining, [14,15] even when a small PanNEN is incidentally discovered. EUS-TA allows preoperative confirmation of the pathological diagnosis and grading of PanNENs, and suitable non-surgical treatments can be selected once unresectable tumors are diagnosed less invasively.

CECT is useful for detecting lesions, assessing vascular invasion, and identifying metastases throughout the body. Therefore, CECT is recommended for tumor detection, staging, resectability assessment, and surveillance during follow-up [16]. PanNETs typically exhibit a hyperattenuation pattern on CECT. Overall, 32–43% of PanNENs contain a non-hyperattenuating mass on CECT [6,17,18]. In our study, 20% of PNETs had a hypoattenuation pattern, likely because cases of PNECs (*n* = 9) were excluded. PanNENs with higher pathological grades tend to show a hypoattenuation pattern on CECT. Patients with PanNET G3 and PanNEC exhibited more frequent hypoattenuation than those with PanNET G1 and G2 [6,7,19]. Consistent with this, our results showed that the hypoattenuation group had a significantly higher pathological grade than the hyperattenuation group. A high pathological grade of PanNETs is more likely to result in metastasis after curative surgery and is associated with a poorer prognosis [20]. In an in vivo model [21] and based on pathological findings [17], poorly differentiated PanNENs demonstrated lower microvascular density than well-differentiated tumors. Necrotic areas of PanNETs showing hypoattenuation on CT indicate microscopic vascular invasion and metastasis [22]. Hypoattenuation tumors may be more likely to develop distant metastases because the incidence of pathological intratumoral necrosis tended to be higher in hypoattenuation groups than in hyperattenuation groups. The hypoattenuation pattern of a tumor may indicate high malignancy [23,24]. Some reports have revealed that the hypoattenuation pattern of PanNENs is associated with a poorer OS rate than the hyperattenuation pattern [5,18,25]. However, all these reports included patients with PanNEC, which requires a different treatment strategy than PanNETs. According to the Japanese guidelines for PanNENs, curative surgery is recommended for resectable PanNETs; however, surgery for PanNEC remains controversial [26]. Deng et al. [27] reported that the median survival for PanNET G3 was 51.3 months, compared with 26.4 months for PanNEC. Therefore, distinguishing between PanNET and PanNEC is essential when evaluating OS, as their treatment strategies and prognoses differ substantially. The novelty of this study is that it demonstrated that hypoattenuated PanNETs, excluding PanNEC, have a poor prognosis. Moreover, PanNET G2 with a hypoattenuation pattern had a worse prognosis than those with a hyperattenuation pattern, although all cases were pathologically classified as G2. The results of our study contribute to short-interval follow-up for patients with hypoattenuated PanNETs, especially those with postoperative recurrence.

The present study has some limitations worth noting. First, it was a retrospective observational study conducted at a single hospital. The CT equipment used for detection was not consistent; however, differences in CT performance likely had little impact, as the tumor enhancement pattern was compared with the normal pancreatic parenchyma. Follow-up periods and treatment strategies varied among the cases. Second, the number of cases was small because PanNETs are rare tumors, which may limit the robustness of analyses. Several CECT imaging factors can also influence prognosis, including tumor size [28,29,30,31], absence of surgery [32,33,34,35], and distant metastasis [36,37,38,39]. Large tumor size has been associated with a poorer prognosis. In this study, tumor size, lack of surgical treatment, and distant metastasis were risk factors for OS in the univariate analysis; however, these factors were not significant in the multivariate analysis. Potential overfitting in the Cox proportional hazards model and confounding bias cannot be entirely excluded because of the limited number of events and sample size. For analyses stratified by pathological grade, the number of PanNET G3 cases and the number of hypoattenuated PanNET G1 cases were small compared with PanNET G2, limiting subgroup analyses. To validate these findings and establish appropriate surveillance algorithms for patients with hypoattenuated PanNETs, larger prospective multicenter studies are required. Third, treatment details other than surgery were not included in the OS analysis. Treatment modalities vary widely, including surgery, chemotherapy, radiotherapy (including PRRT), and local therapies such as TACE. Finally, in non-surgical cases, the pathological grade was determined using EUS-TA. Recent studies have shown that Ki-67 assessment and WHO grading of EUS-TA samples correlate reasonably well with surgical specimens [15,40]. However, the samples obtained by EUS-TA may not always accurately reflect the true tumor grade because of intratumoral heterogeneity and the limited tissue availability [41,42]. Therefore, some degree of misclassification cannot be excluded.

In conclusion, the hypoattenuation pattern during the pancreatic phase of pretreatment CECT was identified as a predictor of poor prognosis in patients with PanNETs, particularly those with PanNET G2. Attenuation characteristics comparing the tumor and pancreatic parenchyma on pretreatment CECT provide useful information for the clinical management of PanNETs.

## Figures and Tables

**Table 1:** Patient characteristics of all patients with pancreatic neuroendocrine tumors.

	Total *n* = 80	Hyperattenuation Group *n* = 64	Hypoattenuation Group *n* = 16	*p*-Value
Age (years), median (range)	64 (14–89)	63 (14–89)	73 (55–86)	0.466
Sex, *n* (%)				1
Male/Female	39/41 (49/51)	31/33 (48/52)	8/8 (50/50)	
Tumor functionality, *n* (%)				0.768
Functional/Non-functional	23/57 (29/71)	18/46 (28/72)	5/11 (31/69)	
Location, *n* (%)				1
Head/Body and tail	46/34 (58/42)	37/27 (58/42)	9/7 (56/44)	
Tumor size(mm), median (range)	18 (6–150)	15 (6–105)	33 (13–150)	**<0.001**
Number of tumors, *n* (%)				0.679
Single/Multiple	71/9 (89/11)	56/8 (88/13)	15/1 (94/6)	
Metastasis, *n* (%)	18 (23)	8 (13)	10 (63)	**<0.001**
Liver	13 (16)	5 (8)	8 (50)	
Lymph node	12 (15)	5 (8)	7 (44)	
Ki-67 LI (%), median (range)	2 (0–50)	1.9 (0–30)	6.5 (13–150)	**0.002**
UICC stage, *n* (%)				**<0.001**
I/II/III/IV	38/23/4/15 (48/29/5/19)	36/19/3/6 (56/30/5/9)	2/4/1/9 (13/25/6/56)	
Resection, *n* (%)	63 (79)	54 (84)	9 (56)	0.894
Curative-intent surgery	57 (71)	51 (80)	6 (38)	**0.033**
Debulking-intent surgery	6 (8)	3 (5)	3 (19)	
2017 WHO classification, *n* (%)				**0.003**
PanNET-G1/G2/G3	45/31/4 (56/39/5)	41/22/1 (64/34/2)	4/9/3 (25/56/19)	
Internal contrast pattern of the tumor, *n* (%)				**0.020**
Homogeneous/Heterogeneous	32/48 (40/60)	30/34 (47/53)	2/14 (13/88)	
Cystic component in the tumor, *n* (%)	13 (16)	10 (16)	3 (19)	0.717
MPD dilation ≥ 5 mm, *n* (%)	6 (8)	2 (3)	4 (25)	**0.013**
Calcification in the tumor, *n* (%)	3 (4)	1 (2)	2 (13)	0.100

Ki-67 LI, Ki-67 labeling index; UICC, Union for International Cancer Control; PanNET, pancreatic neuroendocrine tumor; G, grade; WHO, World Health Organization.

**Table 2:** Univariate and multivariate analyses of risk factors for overall survival in all patients with pancreatic neuroendocrine tumors.

		Univariate Analysis		Multivariate Analysis	
		Hazard Ratio (95% CI)	*p*-Value	Hazard Ratio (95% CI)	*p*-Value
Age (years)	<60 (*n* = 26)	Reference	0.082		
	≥60 (*n* = 54)	3.98 (0.84–18.94)			
Sex	Female (*n* = 41)	Reference	0.176		
	Male (*n* = 39)	2.29 (0.69–7.64)			
Tumor functionality	Functional (*n* = 23)	Reference	0.961		
	Non-functional (*n* = 57)	0.97 (0.29–3.26)			
Location	Head (*n* = 46)	Reference	0.918		
	Body/Tail (*n* = 34)	1.06 (0.34–3.35)			
Liver metastasis	Absence (*n* = 67)	Reference	**0.004**		
	Presence (*n* = 13)	5.40 (1.74–16.77)			
Treatment	Resection (*n* = 63)	Reference	**0.007**		
	Non-resection (*n* = 17)	5.10 (1.56–16.64)			
Tumor size (mm)	<30 (*n* = 60)	Reference	**0.002**		
	≥30 (*n* = 20)	6.73 (2.02–22.40)			
Grade	G1 (*n* = 45)	Reference	**0.007**	Reference	**0.034**
	G2-3 (*n* = 35)	16.75 (2.16–129.90)		9.48 (1.18–76.06)	
Internal contrast pattern	Homogeneous (*n* = 32)	Reference	0.313		
	Heterogeneous (*n* = 48)	1.97 (0.53–7.31)			
Cystic component	Absence (*n* = 67)	Reference	0.280		
	Presence (*n* = 13)	2.07 (0.55–7.77)			
MPD dilation ≥5 mm	Absence (*n* = 74)	Reference	**0.012**		
	Presence (*n* = 6)	4.70 (1.41–15.68)			
Calcification	Absence (*n* = 77)	Reference	**0.024**		
	Presence (*n* = 3)	5.86 (1.26–27.30)			
Contrast pattern	Hyperattenuation (*n* = 64)	Reference	**<0.001**	Reference	**<0.001**
	Hypoattenuation (*n* = 16)	15.31 (4.13–56.73)		9.45 (2.49–35.81)	

CI, confidence interval; G, grade; MPD, main pancreatic duct.

**Table 3:** Patient characteristics of patients with pancreatic neuroendocrine tumors, grade 2.

	Total *n* = 31	Hyperattenuation *n* = 22	Hypoattenuation *n* = 9	*p*-Value
Age (years), median (range)	64 (20–80)	61 (30–80)	73 (20–80)	0.056
Sex, *n* (%)				0.456
Male/Female	17/14 (55/45)	11/11 (50/50)	6/3 (67/33)	
Tumor functionality, *n* (%)				0.639
Functional/Non-functional	7/24 (23/77)	6/16 (27/73)	1/8 (11/89)	
Location, *n* (%)				0.253
Head/Body and Tail	19/12 (61/39)	15/7 (68/32)	4/5 (44/56)	
Tumor size (mm), median (range)	30 (9–150)	22.5 (9–105)	37 (23–150)	**0.042**
Number of tumors, *n* (%)				1
Single/Multiple	28/3 (90/10)	20/2 (91/9)	8/1 (89/11)	
Metastasis, *n* (%)	13 (42)	5 (23)	8 (89)	**0.001**
Liver	10 (32)	3 (14)	7 (78)	
Lymph node	7 (23)	2 (9)	5 (56)	
Ki-67 LI (%), median (range)	5 (3–18)	5 (3–18)	7 (3–15)	0.444
UICC stage, *n* (%)				**0.009**
I/II/III/IV	7/10/3/11 (23/32/10/35)	7/9/2/4 (32/41/9/18)	0/1/1/7 (0/11/11/78)	
Resection, *n* (%)	23 (74)	20 (91)	3 (33)	**0.003**
Curative-intent surgery	19 (61)	17 (77)	1 (11)	0.107
Debulking-intent surgery	4 (13)	3 (14)	2 (22)	
Internal contrast pattern of the tumor, *n* (%)				0.689
Homogeneous/Heterogeneous	9/22 (29/71)	7/15 (32/68)	2/7 (22/78)	
Cystic component in the tumor, *n* (%)	7 (23)	5 (23)	2 (22)	1
MPD dilation ≥5 mm, *n* (%)	5 (16)	2 (9)	3 (33)	0.131
Calcification in the tumor, *n* (%)	2 (6)	0 (0)	2 (22)	0.100

Ki-67 LI, Ki-67 labeling index; UICC, Union for International Cancer Control; MPD, main pancreatic duct.

**Table 4:** Univariate and multivariate analyses of risk factors for overall survival in patients with pancreatic neuroendocrine tumors, grade 2.

		Univariate Analysis		Multivariate Analysis	
		Hazard Ratio (95% CI)	*p*-Value	Hazard Ratio (95% CI)	*p*-Value
Age (years)	<60 (*n* = 11)	Reference	0.110		
	≥60 (*n* = 20)	5.58 (0.68–45.84)			
Gender	Female (*n* = 14)	Reference	0.139		
	Male (*n* = 17)	3.37 (0.68–16.84)			
Tumor functionality	Functional (*n* = 7)	Reference	0.792		
	Non-functional (*n* = 24)	1.24 (0.25–6.26)			
Location	Head (*n* = 19)	Reference	0.255		
	Body/Tail (*n* = 12)	2.25 (0.56–9.12)			
Liver metastasis	Absence (*n* = 21)	Reference	0.106		
	Presence (*n* = 10)	3.27 (0.78–13.78)			
Treatment	Resection (*n* = 23)	Reference	**0.033**		
	Non-resection (*n* = 8)	4.61 (1.13–18.79)			
Ki-67 LI	≤5 (*n* = 17)	Reference	0.194		
	>5 (*n* = 14)	2.91 (0.58–14.54)			
Tumor size (mm)	<30 (*n* = 14)	Reference	0.076		
	≥30 (*n* = 17)	6.67 (0.82–54.37)			
Internal contrast pattern	Homogeneous (*n* = 9)	Reference	0.965		
	Heterogeneous (*n* = 22)	1.04 (0.21–5.14)			
Cystic component	Absence (*n* = 24)	Reference	0.284		
	Presence (*n* = 7)	2.20 (0.52–9.25)			
MPD dilation ≥5 mm	Absence (*n* = 26)	Reference	0.058		
	Presence (*n* = 5)	3.88 (0.96–15.7)			
Calcification	Absence (*n* = 29)	Reference	0.069		
	Presence (*n* = 2)	4.62 (0.89–23.97)			
Contrast pattern	Hyperattenuation (*n* = 22)	Reference	**0.007**	Reference	**0.007**
	Hypoattenuation (*n* = 9)	9.13 (1.83–45.51)		9.13 (1.83–45.51)	

Ki-67 LI, Ki-67 labeling index; MPD, main pancreatic duct; CI, confidence interval.

## Data Availability

The raw data supporting the conclusions of this article will be made available by the authors upon request.

## Supplementary Materials

The following supporting information can be downloaded at: https://www.mdpi.com/article/10.3390/jcm15062252/s1, Supplementary Table S1: Pathological characteristics according to each grade of pancreatic neuroendocrine tumors; Supplementary Table S2: Hounsfield units on computed tomography in the hyperattenuation and hypoattenuation groups; Supplementary Table S3: Comparison of Hounsfield units on computed tomography between the first and last 40 cases; Supplementary Table S4: Incidence of pathological intratumoral necrosis; Supplementary Table S5: Univariate and multivariate analyses of risk factors for overall survival in patients who underwent surgery; Supplementary Table S6: Estimation of overall survival and progression free survilval at 1, 3, 5, and 10 years by Kaplan–Meier analysis.

## Abbreviations

The following abbreviations are used in this manuscript:

CECT	contrast-enhanced computed tomography
PanNET	pancreatic neuroendocrine tumor
OS	overall survival
G	grade
PanNENs	pancreatic neuroendocrine neoplasms
WHO	World Health Organization
PanNEC	pancreatic neuroendocrine carcinoma
EUS-TA	endoscopic ultrasound-guided tissue acquisition
UICC	Union for International Cancer Control
HU	Hounsfield units
PRRT	peptide receptor radionuclide therapy
TACE	transcatheter arterial chemoembolization
HR	hazard ratio
CI	confidence interval
PFS	progression-free survival
