# Supplementary material for: Hypoattenuation Pattern on Contrast-Enhanced Computed Tomography Predicts Poor Prognosis in Patients with Pancreatic Neuroendocrine Tumors
Source: J Clin Med. 2026 Mar 16;15(6):2252. doi: 10.3390/jcm15062252 (PMC13026872; doi:10.3390/jcm15062252)
Supplement: Supplementary file 1 [file jcm-15-02252-s001.zip › jcm-4058878-supplementary.pdf]

Journal of
Clinical Medicine

MDPI

Supplementary Table S1. Pathological characteristics according to each grade of pancreatic neuroendocrine tumors.

	Grade of pancreatic neuroendocrine tumors*		
	**G1**	**G2**	**G3**
Ki-67 LI (%), median (range)	1.0 (0-2.8) (n=45)	5.0 (3.0-18.0) (n=31)	35.0 (30.0-50.0) (n=4)
Mitotic rate(/2mm^3), median (range)	0 (0-2) (n=44)	1 (0-9) (n=27)	15 (2-28) (n=2)
Necrosis, % (n)	5.0 (1/20)	30.8 (4/13)	0.0 (0/1)
SSTR2, % (n)	83.8 (31/37)	92.3 (24/26)	50.0 (1/2)
Chromogranin A, % (n)	97.6 (41/42)	89.3 (25/28)	100 (2/2)
Synaptophysin, % (n)	95.2 (40/42)	92.3 (24/26)	100 (2/2)

Ki-67 LI, Ki-67 labeling index; WHO, World Health Organization; G, grade; SSTR2, Somatostatin Receptor type2

* Seventeen patients were diagnosed with endoscopic ultrasound-guided tissue acquisition (EUS-TA), and two were diagnosed during a second EUS-TA session. One patient underwent EUS-TA using a 25-gauge biopsy needle in the first session but did not receive a diagnosis. A 22-gauge biopsy needle was used in the second session. In another case, a 22-gauge aspiration needle was used in the first session, but it did not yield a definitive diagnosis. Another 22-gauge aspiration needle was used in the second session.

Supplementary Table S2. Hounsfield units on computed tomography in the hyperattenuation and hypoattenuation groups.

	**Total (n = 80)**	**Hyperattenuation group (n = 64)**	**Hypoattenuation group (n = 16)**	***p*-Value**
Tumor, HU (range)	205.12 (61.82–440.86)	217.31 (108.25–440.86)	118.36 (61.82–157.22)	**<0.001**
Pancreatic parenchyma, HU (range)	157.29 (95.24–213.69)	157.29 (95.24–205.28)	158.44 (105.66–213.69)	0.732
HU ratio (tumor to pancreatic parenchyma)	1.31 (0.38–3.32)	1.43 (1.00–3.32)	0.75 (0.38–0.99)	**<0.001**

HU, Hounsfield units

Supplementary Table S3. Comparison of Hounsfield units on computed tomography between the first and last 40 cases.

		First 40 cases	Last 40 cases	*p*-Value
Total cases (n = 80)	Tumor, HU (range)	205.12 (79.81–341.72)	204.33 (61.82–440.86)	0.815
	Pancreatic parenchyma, HU (range)	154.00 (95.24–213.69)	160.53 (105.66–198.85)	0.312
	HU ratio (tumor to pancreatic parenchyma)	1.31 (0.59–3.32)	1.30 (0.38–2.64)	0.943
Hyperattenuation group (n = 64)<br>First 40 cases (n = 32)<br>Last 40 cases (n = 32)	Tumor, HU (range)	215.50 (108.25–341.72)	217.31 (157.49–440.86)	0.704
	Pancreatic parenchyma, HU (range)	154.00 (95.24–205.28)	159.92 (113.52–198.85)	0.367
	HU ratio (tumor to pancreatic parenchyma)	1.42 (1.00–3.32)	1.44 (1.02–2.64)	0.847
Hypoattenuation group (n = 16)<br>First 40 cases (n = 8)<br>Last 40 cases (n = 8)	Tumor, HU (range)	132.70 (79.81–156.92)	109.22 (61.82–157.22)	0.382
	Pancreatic parenchyma, HU (range)	148.54 (134.16–213.69)	164.87 (105.66–179.91)	0.574
	HU ratio (tumor to pancreatic parenchyma)	0.76 (0.59–0.99)	0.75 (0.38–0.96)	0.505

HU, Hounsfield units

Supplementary Table S4. Incidence of pathological intratumoral necrosis.

		Hyperattenuation group	Hypoattenuation group	*p*-Value
Total cases (n=34)	Necrosis, % (n)	10.0 (3/30)	50.0 (2/4)	0.094
Total surgery cases (n=32)	Necrosis, % (n)	10.3 (3/29)	66.7 (2/3)	0.057

Supplementary Table S5. Univariate and multivariate analyses of risk factors for overall survival in patients who underwent surgery.

		Univariate analyses		Multivariate analyses	
		**Hazard ratio (95% CI)**	***p*-Value**	**Hazard ratio (95% CI)**	***p*-Value**
Age (years)	＜60 (n=23)	Reference	0.329		
	≥60 (n=40)	2.32 (0.43–12.50)			
Gender	Female (n=31)	Reference	0.173		
	Male (n=32)	3.14 (0.60–16.34)			
Tumor functionality	Functional (n=20)	Reference	0.229		
	Non-functional (n=43)	0.40 (0.09–1.79)			
Location	Head (n=40)	Reference	0.214		
	Body/Tail (n=23)	0.26 (0.03–2.18)			
Liver metastasis	Absence (n=58)	Reference	**0.010**		
	Presence (n=5)	7.25 (1.62–32.44)			
Treatment	Curative (n=57)	Reference	**0.002**	Reference	
	Debulking (n=6)	10.56 (2.35–47.43)		6.02 (1.17-31.14)	**0.032**
Tumor size (mm)	<30 (n=51)	Reference	0.117		
	≥30 (n=12)	3.32 (0.74–14.85)			
Grade	G1 (n=38)	Reference	**0.024**		
	G2-3 (n=25)	11.50 (1.38-95.92)			
Internal contrast pattern	Homogeneous (n=25)	Reference	0.442		
	Heterogeneous (n=38)	1.91 (0.37–9.99)			
Cystic component	Absence (n=53)	Reference	0.833		
	Presence (n=10)	1.26 (0.15–10.62)			
MPD dilation ≥5 mm	Absence (n=57)	Reference	**0.002**		
	Presence (n=6)	10.26 (2.28–46.08)			
Calcification	Absence (n=61)	Reference	0.193		
	Presence (n=2)	4.17 (0.49–35.82)			
Contrast pattern	Hyperattenuation (n=54)	Reference	**0.003**	Reference	**0.046**
	Hypoattenuation (n=9)	9.71 (2.16–43.75)		5.38 (1.03–28.08)	

MPD, main pancreatic duct; CI, confidence interval.

Supplementary Table S6. Estimation of overall survival and progression free survilval at 1, 3, 5, and 10 years by Kaplan – Meier analysis.

			**yeas**				***p*-Value**
			**1**	**3**	**5**	**10**	
Total cases (n = 80)	OS	Hyperattenuation group (n = 64)	100%	100%	97.6%	82.9%	**<0.001**
		Hypoattenuation group (n = 16)	75.0%	61.1%	45.8%	30.6%	
	PFS	Hyperattenuation group (n = 64)	98.2%	96.1%	91.1%	87.6%	**<0.001**
		Hypoattenuation group (n = 16)	50.0%	41.7%	41.7%	41.7%	
Total surgery cases (n=63)	OS	Hyperattenuation group (n = 54)	100%	100%	97.3%	82.7%	**<0.001**
		Hypoattenuation group (n = 9)	88.9%	88.9%	63.5%	31.7%	
	PFS	Hyperattenuation group (n = 54)	98.0%	95.5%	92.9%	88.6%	**<0.001**
		Hypoattenuation group (n = 9)	66.7%	53.3%	53.3%	53.3%	
G2 cases (n=31)	OS	Hyperattenuation group (n = 22)	100%	100%	92.3%	76.9%	**0.001**
		Hypoattenuation group (n = 9)	77.8%	53.3%	40.0%	0%	
	PFS	Hyperattenuation group (n = 22)	95.0%	88.2%	72.8%	60.6%	**0.001**
		Hypoattenuation group (n = 9)	33.3%	33.3%	33.3%	0%	
G2 surgery cases (n=23)	OS	Hyperattenuation group (n = 20)	100%	100%	97.3%	82.7%	**0.063**
		Hypoattenuation group (n = 3)	88.9%	88.9%	63.5%	31.7%	
	PFS	Hyperattenuation group (n = 20)	94.4%	86.6%	78.7%	63.0%	**0.005**
		Hypoattenuation group (n = 3)	33.3%	33.3%	0%	0%	

OS, overall survival; G, grade; PFS, progression free survival
